# Psychometric characteristics of the career adapt-abilities scale in Thai undergraduate students: a multiple indicators multiple causes model

**DOI:** 10.3389/fpsyg.2024.1338401

**Published:** 2024-01-31

**Authors:** Buratin Khampirat

**Affiliations:** Institute of Social Technology, Suranaree University of Technology, Nakhon Ratchasima, Thailand

**Keywords:** career adaptability, career counseling, MIMIC model, reliability, validity, psychometrics, Thai students

## Abstract

**Objective:**

This study aimed to evaluate the psychometric properties of the Career Adapt-abilities Scale (CAAS) and analyze the relationships between sociodemographic variables and career adaptability using the MIMIC model with DIF.

**Methods:**

In this cross-sectional study, the CAAS, consisting of four sub-scales—concern, control, curiosity, and confidence—each comprising six items, was administered to 620 undergraduate students from 34 universities in Thailand. Among the participants, women constituted 66.77%, while men accounted for 32.58% (Mean Age = 20.33). To explore the influence of sociodemographic characteristics on specific CAAS item responses, a Multiple-Indicators, Multiple-Causes model with Differential Item Functioning (MIMIC-model with DIF) was employed.

**Results:**

The findings revealed robust internal consistency and reliability in the CAAS. Both the four-factor and second-order factor models exhibited excellent fit indices, emphasizing the significance of each item within the measure. Sociodemographic characteristics did not demonstrate a significant direct effect on the four CAAS subscales, it is worth noting the exception regarding paternal education. Paternal education was found to have a statistical significance impact on two specific CAAS items: “looking for opportunities to grow as a person” and “becoming curious about new opportunities.”

**Conclusion:**

These findings underscore the intricate influence of paternal education on specific aspects of career adaptability, suggesting that certain dimensions of career adaptability may be shaped by factors extending beyond the conventional sociodemographic variables examined in this study. Future research could delve deeper into the complex interplay of sociodemographic factors and individual attributes to provide a more holistic perspective on career adaptability in Thailand.

## 1 Introduction

The digital era has resulted in rapid transformations across various dimensions, while the COVID-19 pandemic has imposed significant economic, livelihood, and occupational challenges (Watermeyer et al., 2021). Notably, the pandemic triggered a substantial increase in the unemployment rate (Kunchai et al., 2021). Modern organizations increasingly require individuals adept at working with contemporary technology (Chonsalasin and Khampirat, 2022). Graduating students faced considerable difficulty securing employment, impacting their career prospects (ILO, 2020). In these dynamic times, career adapt-abilities emerge as a critical factor in working and getting a job (Savickas et al., 2009).

Careers adapt-abilities are intrinsic resources that enable individuals to deal with tasks and job-related challenges (Savickas and Porfeli, 2012). This adaptability enables individuals to reconcile their internal needs with external challenges such as conforming to age-based social norms, navigating job changes, and dealing with difficult situations like contract breaches, unemployment, career change, plant closures, or workplace accidents (Xu et al., 2020; Savickas, 2022).

Savickas and Porfeli (2012) and Savickas (2022) developed the Career Adapt-Abilities Scale (CAAS) to aid individuals in preparing for their future professional roles. This scale evaluates individuals across four dimensions—concern, control, curiosity, and confidence—forming a multidimensional construct. Past research has highlighted positive correlations between CAAS scores and work motivation (Leong et al., 2022), happiness at work (Yang et al., 2019; Rasheed et al., 2020), ambidexterity, service performance (Affum-Osei et al., 2021). as well as dimensions like quality of life, well-being (Leong et al., 2022), resilience, life satisfaction (Santilli et al., 2020; Di Maggio et al., 2022), pathological characteristics (Gonçalves et al., 2021), and life meaning (Sou et al., 2021).

While the CAAS has been translated and validated in various cultural contexts globally and were confirmed the psychometric properties of the instrument in term of construct validity and reliability, establishing its appropriateness for application across diverse contexts (Duangsao et al., 2021; Kondratyuk et al., 2021; Ghosh et al., 2022; Leong et al., 2022; Soares et al., 2022; Song et al., 2023). Nevertheless, based on available information, its utilization in the Thai context has not been thoroughly investigated within the broader student population, with the exception of the study by Assapun and Sophonhiranrak (2018) and Duangsao et al. (2021), which focused solely on Bachelor students in the capital and surrounding areas. Especially the testing using multiple-indicators, multiple-causes model with differential item functioning (MIMIC-model with DIF) still untested. This study therefore aimed to (a) evaluate the psychometric properties of the CAAS in a Thai context, and (b) analyze the relationships between sociodemographic variables and career adaptability using the MIMIC model with DIF.

## 2 Literature review

### 2.1 Career situation of a new graduate in Thailand

For a recent graduate in Thailand, the career landscape is both promising and challenging. The Kingdom of Thailand, known for its vibrant culture and dynamic economy, offers diverse opportunities for young professionals (Pongsin et al., 2023). However, the competition is fierce, and navigating the job market requires a combination of education, skills, and networking. New graduates have a high rate of employment that does not match their field of study (Sa-Nguanmanasak and Khampirat, 2019). Upon graduation, many Thai students face the decision of whether to pursue further studies or enter the workforce directly (Sa-Nguanmanasak and Khampirat, 2019). The job market in Thailand is characterized by a demand for skilled professionals in sectors such as technology, digital marketing, tourism, finance, and healthcare. Technical skills and English proficiency are often valuable assets, particularly with the growing globalization of businesses in the country (Khampirat, 2021; It-ngam et al., 2023). Despite this, Thai students continue to encounter issues related to skills mismatch and low proficiency in the English language (Sa-Nguanmanasak and Khampirat, 2019; Bamrungsin and Khampirat, 2022).

In recent years, there has been a growing emphasis on entrepreneurship in Thailand (Putri et al., 2023). Some graduates choose to start their own businesses, leveraging their education and skills to contribute to the country’s economic landscape. The Thai government has also implemented initiatives to support startups, providing resources and incentives for young entrepreneurs (Somwethee et al., 2023). However, challenges persist, including issues like underemployment and a gap between the skills obtained through education and those required by the job market (Kunchai et al., 2021). Navigating these challenges requires adaptability, continuous learning, and a proactive approach to career development.

Today, the surge in youth unemployment is a pressing concern, accompanied by a notable transformation in the structure and dynamics of employment. Consequently, many young individuals find themselves engaged in occupations misaligned with their academic pursuits, resulting in skill disparities and remuneration that falls short of their qualifications. In response to these challenges, Thailand is actively undertaking initiatives to provide ongoing training to bolster skill sets. Despite these efforts, a lingering sense of insecurity persists among individuals regarding their competencies and knowledge (Kunchai et al., 2021). Therefore, the development of career adaptability is essential. Because the ability to anticipate obstacles and opportunities, adapt to the environment, explore various career options, and create a plan is crucial for achieving personal and professional goals. Nonetheless, it is essential to have an accurate tool to evaluate adaptability. Only by doing so can people gain the confidence to navigate unexpected challenges and pursue their desired outcomes (Soares et al., 2022).

### 2.2 Historical context of the psychometric properties of CAAS

The CAAS, developed by Savickas and Porfeli (2012), was designed to measure an individual’s ability to adapt to career changes. Rooted in the career construction theory (CCT) (Savickas, 2005), CAAS posits that individuals construct their careers through adaptation to changing circumstances. The scale consists of four dimensions: concern, control, curiosity, and confidence. Concern reflects preparedness for one’s future career, prompting planning and readiness for professional scenarios (Prasad et al., 2021). Control signifies an individual’s commitment to developing and steering their profession, involving self-control, persistence, and proactive decision-making (Savickas, 2022). Curiosity entails exploring diverse career options, engaging with the job market, defining future self, and contemplating potential career roles (Matijaš and Seršić, 2021). Confidence mirrors an individual’s belief in their ability to tackle challenges, achieve goals, and resolve workplace difficulties, indicating conviction in accomplishing objectives despite obstacles (Di Maggio et al., 2022). Variation in CAAS scores can be possible due to both systematic variation due to characteristics of the measurement method and random variation due to differences in individual characteristics or measurement situations. From the published literature, a number of studies have examined the psychometric properties of the CAAS in the areas of (a) test–retest reliability, (b) internal consistency, (c) cross-cultural reliability, and (d) construct validity studies.

#### 2.2.1 Test–retest reliability studies

A fundamental aspect of reliability is the test–retest reliability, which illuminates the stability of scores over time. Longitudinal assessments of the CAAS, conducted by researchers such as Tolentino et al. (2014), Di Maggio et al. (2015), and Sidiropoulou-Dimakakou et al. (2018), involved measuring participants at two different points. The results, as indicated by Pearson’s r, demonstrated a commendable stability in CAAS scores, affirming its reliability in capturing the enduring aspects of career adaptability.

#### 2.2.2 Internal consistency studies

Another facet of reliability is internal consistency, which evaluates the homogeneity of items within each subscale of CAAS using Cronbach’s alpha. Researchers, including Öncel (2014), McKenna et al. (2016), and Prasad et al. (2021) conducted comprehensive studies analyzing the internal consistency of CAAS dimensions. The results indicated high levels of internal consistency of CAAS dimensions. The outcomes revealed robust internal consistency, with Cronbach’s alpha values ranging between 0.79 to 0.95, providing evidence for the coherence and reliability of the scale in assessing specific facets of career adaptability.

#### 2.2.3 Cross-cultural reliability

In the pursuit of establishing CAAS as a globally applicable instrument, researchers have explored its reliability across diverse cultural contexts. Studies, for example, Porfeli and Savickas (2012), Lee et al. (2021), conducted a cross-cultural study involving participants from different samples. The outcomes not only supported the reliability of CAAS across different cultures but also underscored its adaptability as a reliable measure in diverse socio-cultural settings.

#### 2.2.4 Construct validity studies

Crucial for ensuring an instrument measure what it purports to measure, construct validity has been a central focus in CAAS research. Studies, such as those by Savickas and Porfeli (2012), Tien et al. (2014), Duangsao et al. (2021), Soares et al. (2022), and Yang et al. (2023), employed confirmatory factor analyses and other methodologies to validate the theoretical structure of CAAS. This research offers empirical support for its robust construct validity across diverse cultural and educational contexts.

The exploration of psychometric properties of the CAAS has been a dynamic and ongoing process. With a foundation in career construction theory, CAAS has demonstrated its reliability and validity through various studies, providing researchers and practitioners with a robust tool for assessing career adaptability. As scholars continue to delve into advanced methodologies and diverse samples, the understanding of CAAS and its applicability in different contexts will deepen.

## 3 Methods

### 3.1 Participants

This study was a cross-sectional study. The determination of the sample size for this research was based on an approach for confirmatory factor analysis (CFA), established through several essential statistical criteria. Common guidelines initially recommend a base size of 200 participants, then suggest a ratio of 5–10 participants for each estimated parameter, ensuring representativeness of the target population. Furthermore, the desired statistical power was set at 0.95, as calculated using the G*Power program. To reduce selection bias, this study employed a stratified random sampling method. The participants were 620 Thai undergraduate students (32.58% female and 66.77% male); we excluded from the analyses four participants who did not provide a response regarding their gender. The participants ages ranged between 18 and 35 years (Mean Age = 20.33, *SD* = 1.83), and correspondingly, they spanned a range of years of study (43.23% were freshmen, and 16.45% were seniors) and majors; more than half of the students were majoring in the science and technology program (51.61%) and the rest in social sciences. In contrast, most survey respondents’ parents had less than a bachelor’s degree education (Table 1).

**Table 1:** Participants’ sociodemographic profiles (*N* = 620).

Variable	Code description	Category	*N*	%
Gender	1 = female (reference group) 0 = otherwise	Male	202	32.58
		Female	414	66.77
		N/A	4	0.65
Age	Ranged from 18 to 35 years old (Mean Age = 20.33, *SD* = 1.83)	<= 20 years old	330	53.23
		21–23 years old	226	36.45
		> = 24 years old	25	4.03
		N/A	39	6.29
Major		Management/Business	70	11.29
		Education	143	23.06
		Information Technology	195	31.45
		Communication Arts	62	10.00
		Health Science	17	2.74
		Engineering	73	11.77
		Agricultural Science	7	1.13
		Computer	24	3.87
		Science	4	0.65
		Social Science	25	4.03
Program	1 = program in social science (reference group) 0 = otherwise	Science &Technology	320	51.61
		Social Science	300	48.39
Year of study	1 = freshman (reference group) 0 = otherwise	Freshman	268	43.23
		Sophomore	85	13.71
		Junior	165	26.61
		Senior	102	16.45
Type of University	1 = Research university (reference group) 0 = otherwise	Other universities	318	51.29
		Research university	302	48.71
Region of University	1 = National Capital Region (reference group) 0 = otherwise	National Capital Region	75	12.10
		Other	545	87.90
Paternal education	1 = bachelor’s degree and higher (reference group) 0 = otherwise	Kindergarten	5	0.81
		Elementary school	158	25.48
		Lower secondary	69	11.13
		Upper secondary	138	22.26
		Training for career	65	10.48
		High vocational diploma	106	17.10
		Bachelor’s degree	24	3.87
		Master’s degree	2	0.32
		Doctorate degree	47	7.58
		N/A	6	0.97
Maternal education	1 = bachelor’s degree and higher (reference group) 0 = otherwise	Kindergarten	7	1.13
		Elementary school	169	27.26
		Lower secondary	87	14.03
		Upper secondary	127	20.48
		Training for career	57	9.19
		High vocational diploma	112	18.06
		Bachelor’s degree	27	4.35
		Master’s degree	1	0.16
		Doctorate degree	28	4.52
		N/A	5	0.81

### 3.2 Measures

Career adaptability was assessed using the international version of the CAAS self-assessment questionnaire, as developed by Savickas and Porfeli (2012). The scale comprises 24 items distributed across four subscales: concern, control, curiosity, and confidence. Respondents rated each item on a 5-point Likert-type scale, ranging from 1 (least or not strong) to 5 (strongest). Surveys with more than one missing item in any subscale were excluded from the analysis.

### 3.3 Procedure

Both online and paper-and-pencil questionnaires were distributed to students across 34 universities spanning diverse regions in Thailand. This data collection process extended over 10 months, from August 2021 to May 2022. On average, participants dedicated 10–15 min to complete the survey. To ensure that this study participant is representative of the targeted population and to minimize bias in the results, the inclusions criteria were who are willing to participate in the study and must be Thai undergraduate students currently enrolled in universities. Exclusion criteria comprised participants who are unwilling or unable to provide informed consent and emotional conditions that may affect study results. Participants’ identities and information were kept private and confidential. Prior to data collection, participants were asked for consent, and no compensation was provided for their involvement. This study was reviewed and approved by the ethics committee for research involving human subjects at Suranaree University of Technology (EC-63-85).

### 3.4 Analyses

After data cleansing, the normality of variable distributions was assessed using statistical tests such as skewness (*SK*), kurtosis (*KU*), and the Mardia’s coefficients, alongside visual tools. As indicated in Table 2, the kurtosis and skewness values were below an absolute value of 2.0 (*SK* < 2) and 7.0 (*KU* < 7) respectively. The *p*-values for Mardia’s coefficients were greater than 0.05, suggesting that the data followed a normal distribution (Kim, 2013; Khampirat, 2021). Subsequently, outlier detection was conducted using methods like the Interquartile Range (IQR) rules and Z-scores, which revealed no outlier data points. Descriptive statistics was conducted using SPSS 26.0 and employed Cronbach’s alpha (α) to evaluate internal reliability. For psychometric scales, reliability reflects random error. That means, if the reliability coefficient is 0.80, it signifies a 36% error variance (random error) in the scores (calculated as 0.80 × 0.80 = 0.64; therefore, 1.00–0.64 = 0.36) (Kline, 1994). Pearson’s correlation (r) matrix was computed to examine the associations among the variables (Cohen et al., 2002).

**Table 2:** Career adapt-abilities scale: items, descriptive statistics, and internal consistencies.

Factor/Items	Code	*M*	*SD*	*SK*	*KU*	CITC
**Concern (α = 0.918)**						
1 Thinking about what my future will be like	Adap1	4.19	0.95	−1.11	0.74	0.75
2 Realizing that today’s choices shape my future	Adap2	4.35	0.82	−1.28	1.46	0.70
3 Preparing for the future	Adap3	3.97	0.96	−0.58	−0.36	0.80
4 Becoming aware of the educational and vocational choices that I must make	Adap4	4.09	0.94	−0.92	0.47	0.81
5 Planning how to achieve my goals	Adap5	4.03	0.91	−0.72	0.18	0.79
6 Concerned about my career	Adap6	4.22	0.88	−0.99	0.50	0.77
**Control (α = 0.876)**						
7 Keeping upbeat	Adap7	4.31	0.88	−1.35	1.73	0.64
8 Making decisions by myself	Adap8	4.07	0.92	−0.86	0.47	0.69
9 Taking responsibility for my actions	Adap9	4.38	0.77	−1.22	1.33	0.68
10 Sticking up for my beliefs	Adap10	4.21	0.84	−1.00	0.91	0.74
11 Counting on myself	Adap11	4.25	0.95	−1.19	0.86	0.73
12 Doing what’s right for me	Adap12	4.35	0.79	−1.03	0.44	0.63
**Curiosity (α = 0.883)**						
13 Exploring my surroundings	Adap13	4.24	0.84	−0.93	0.50	0.65
14 Looking for opportunities to grow as a person	Adap14	4.33	0.79	−1.05	0.89	0.70
15 Investigating options before making a choice	Adap15	4.33	0.78	−1.01	0.72	0.69
16 Observing different ways of doing things	Adap16	4.17	0.83	−0.76	0.12	0.76
17 Probing deeply into questions that I have	Adap17	4.04	0.88	−0.65	0.08	0.67
18 Becoming curious about new opportunities	Adap18	4.28	0.80	−1.06	1.03	0.70
**Confidence (α = 0.898)**						
19 Performing tasks efficiently	Adap19	4.00	0.84	−0.47	−0.32	0.74
20 Taking care to do things well	Adap20	4.31	0.77	−0.97	0.83	0.71
21 Learning new skills	Adap21	4.15	0.81	−0.73	0.21	0.72
22 Working up to my ability	Adap22	4.30	0.82	−0.99	0.44	0.69
23 Overcoming obstacles	Adap23	4.00	0.90	−0.56	−0.28	0.75
24 Solving problems	Adap24	3.97	0.86	−0.44	−0.40	0.75
**Career adaptability (α = 0.919)**						
Concern		4.14	0.767	−1.045	1.310	0.784
Control		4.26	0.676	−1.127	1.601	0.815
Curiosity		4.23	0.652	−0.930	1.427	0.835
Confidence		4.12	0.680	−0.639	0.307	0.830

SE of KU = 0.196; SE of SK = 0.098.

To assess the construct validity of the baseline models of the CAAS, confirmatory factor analyses (CFA) were conducted, and the degree of fit was calculated using maximum likelihood estimation in the Mplus 8.13 statistical package. Model fit was evaluated based on several goodness-of-fit indices, including χ2/df, the comparative fit index (CFI), the Tucker–Lewis index (TLI), the root mean square error of approximation (RMSEA), and the standardized root mean squared residual (SRMR). Adequate fit was determined if χ2/df ≤ 3 (Kline, 2016), RMSEA and SRMR were below 0.08, and CFI and TLI were ≥ 0.90 (Hu and Bentler, 1998).

**Figure 1:**
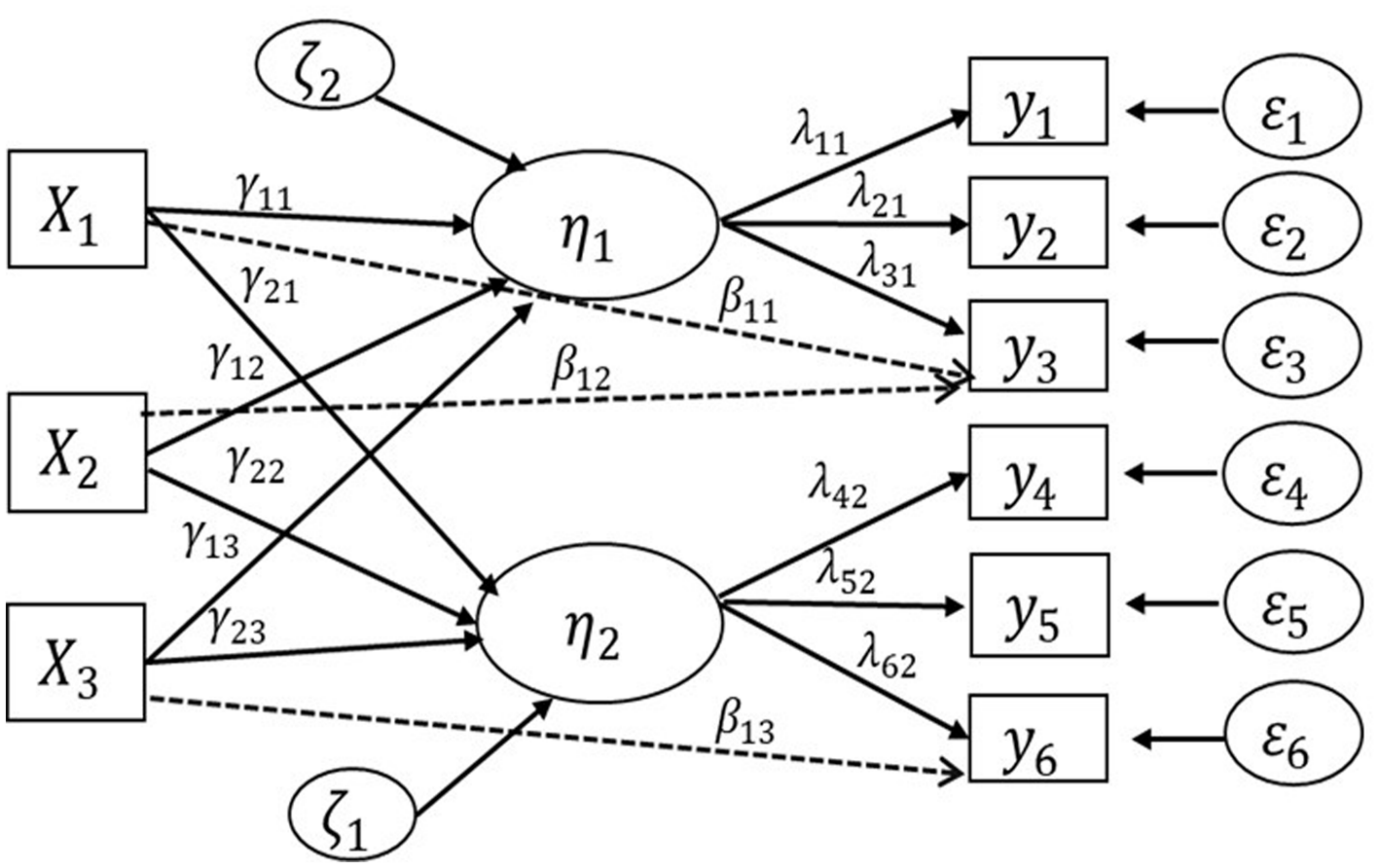
MIMIC model with DIF.

Upon achieving a satisfactory fit for the baseline CFA model, a MIMIC model (depicted in Figure 1) was implemented to assess the measurement invariance of the CAAS across different sociodemographic variables and the four factors. Specifically, the exploration of construct validity involved estimating the impact of potential covariates on the four CAAS subscales and the DIF effect. The initial test involved the four-factor MIMIC model without DIF, followed by the detection of DIF to analyze the direct path of covariates on specific item responses (indicators). The sociodemographic variables incorporated as covariates in the MIMIC model included gender (with female as the reference group), age (with at least 20 years as the reference group), university region (with the national capital region as the reference group), program (with social science as the reference group), and paternal education (with a bachelor’s degree and higher as the reference group).

In Figure 1, an illustrative MIMIC model is presented, featuring three observed causes (X1, X2, X3) and six observed indicators (y1, y2, y3, y4, y5, y6) for the latent variables (η1 and η2). The ovals depict the latent factors, while the rectangles denote the covariate (on the left) and items (on the right). The solid and dashed lines from covariates to latent factors and items symbolize regression paths, illustrating the influence of predictors on latent factors and items, respectively. The arrows connecting the latent factors to each item signify their standard loadings (λ) (Kunchai et al., 2021).

## 4 Results

### 4.1 Descriptive statistics

Table 2 presents the CAAS-Thai items for each factor and their internal consistency reliabilities. We computed univariate statistics for all the observed indicator items from the CAAS, and the kurtosis (−1.275 to −0.443) and skewness (−0.395 to 1.729) demonstrated that the 24 CAAS-Thai items conformed to the CFA assumptions. Students’ scores were the highest for the following items: Adap2 (*M* = 4.353, *SD* = 0.823) for concern, Adapt9 (*M* = 4.381, *SD* = 0.773) for control, Adapt14 (*M* = 4.329, *SD* = 0.789) for curiosity, and Adapt20 (*M* = 4.306, *SD* = 0.773) for confidence. They gave themselves the lowest ratings for Adapt3 (*M* = 3.971, *SD* = 0.955), Adapt8 (*M* = 4.073, *SD* = 0.920), Adapt17 (*M* = 4.044, *SD* = 0.882), and Adapt24 (*M* = 3.968, *SD* = 0.860) for concern, control, curiosity, and confidence, respectively.

The mean scores for the Thai CAAS were concern = 4.14, control = 4.26, curiosity = 4.23, and confidence = 4.12, all higher than the international mean. The kurtosis and skewness for the four CAAS-Thai subscales ranged, respectively, from −1.127 to −0.639 and 0.307 to 1.601: The subscales conform to the assumptions of correlation-based statistics for this sample. In addition, the standard errors of kurtosis and skewness were well within a tolerable range for assuming a normal distribution of the data.

### 4.2 Psychometric properties of CAAS-Thai

Cronbach’s alpha (*α*) for the four factors indicated robust internal consistency, with values ranging from 0.876 to 0.918. The overall CAAS-Thai demonstrated even higher internal consistency with *α* = 0.961, surpassing the values for the individual subscales and the composite subscale (*α* = 0.919). Specifically, the alpha values for the individual subscales were as follows: concern = 0.918, control = 0.876, curiosity = 0.883, and confidence = 0.898. The correlation coefficients between the four subscales were statistically significant at *p* < 0.01, ranging from 0.738 to 0.814.

Table 3 provides comprehensive fit indices for the measurement and MIMIC models of the CAAS, affirming the adequacy of these models in capturing career adaptability within the Thai context. The first-order four-factor model, examining the individual dimensions of career adaptability, demonstrated robust fit indices: *χ^2^* = 403.418, *df* = 230, *p* < 0.001, *χ^2^/df* = 1.754, CFI = 0.976, TLI = 0.972, RMSEA = 0.037 (95% CI: 0.031, 0.043). Similarly, the second-order factor model, which synthesizes these dimensions into an overarching construct, exhibited favorable fit indices: *χ^2^* = 413.481, *df* = 231, *p* < 0.001, *χ^2^/df* = 1.790, CFI = 0.975, TLI = 0.970, and RMSEA = 0.038 (95% CI: 0.032, 0.044). These results signify the models’ capability to effectively measure career adaptability among Thai undergraduate students.

**Table 3:** Model fit indices: measurement model and MIMIC model.

Models	*χ^2^*	*df*	p	*χ^2^/df*	CFI	TLI	RMSEA (95% CI)	SRMR
**Measurement model**								
Four-factor model	403.418	230	0.0000	1.754	0.976	0.972	0.037 (0.031–0.043)	0.033
Second-order factor model	413.481	231	0.0000	1.790	0.975	0.970	0.038 (0.032–0.044)	0.035
**MIMIC model**								
MIMIC model with DIF-18 items	888.168	278	0.0000	3.195	0.918	0.883	0.072 (0.066–0.077)	0.155

The MIMIC model with Differential Item Functioning (DIF) for 18 items also demonstrated a strong fit: *χ^2^* = 888.168, *df* = 278, *p* < 0.001, *χ^2^/df* = 3.195, CFI = 0.918, TLI = 0.883, and RMSEA = 0.072 (95% CI: 0.066, 0.077). This model allowed for an exploration of the influence of sociodemographic factors on both the overall CAAS score and specific item responses.

**Figure 2:**
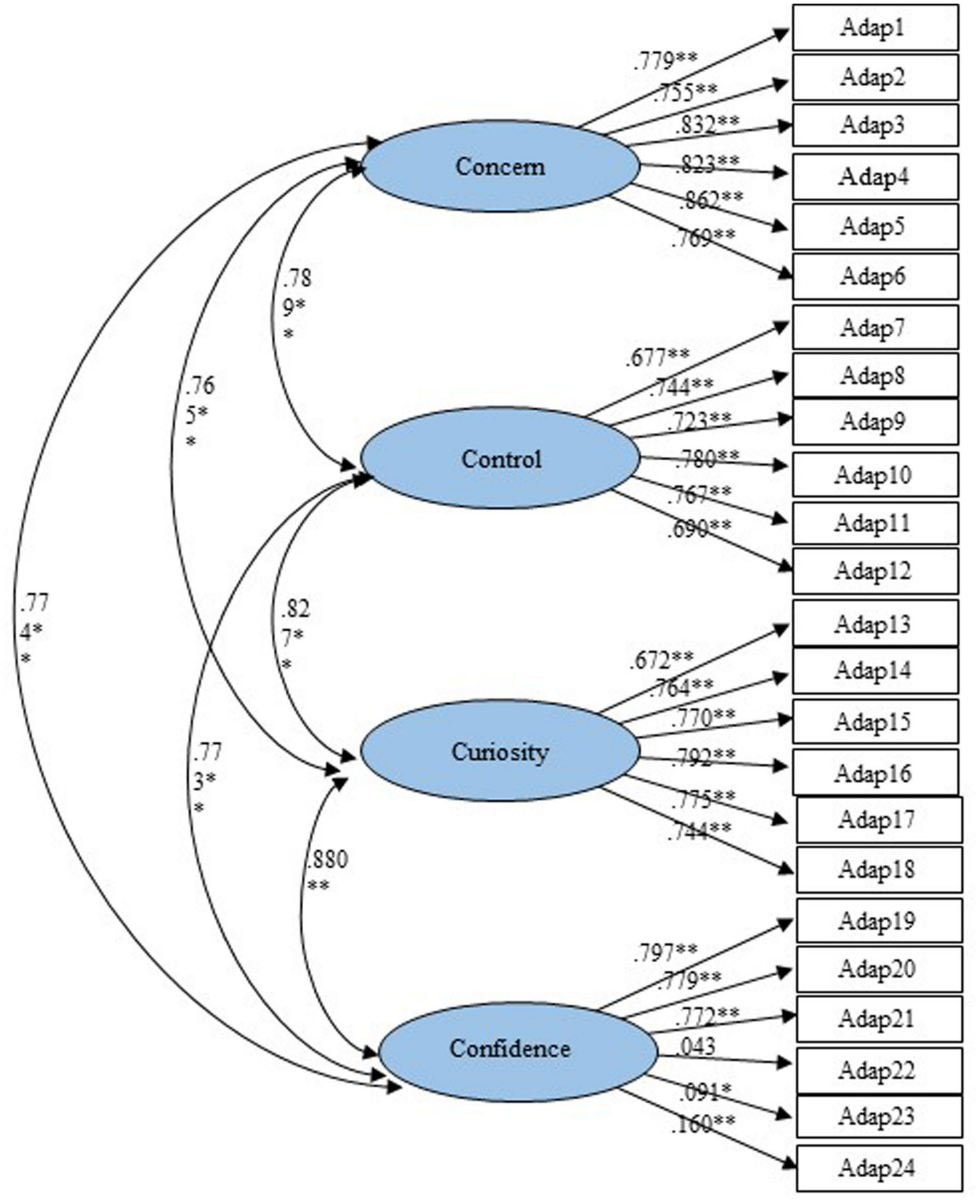
Four-factor CFA model.

**Figure 3:**
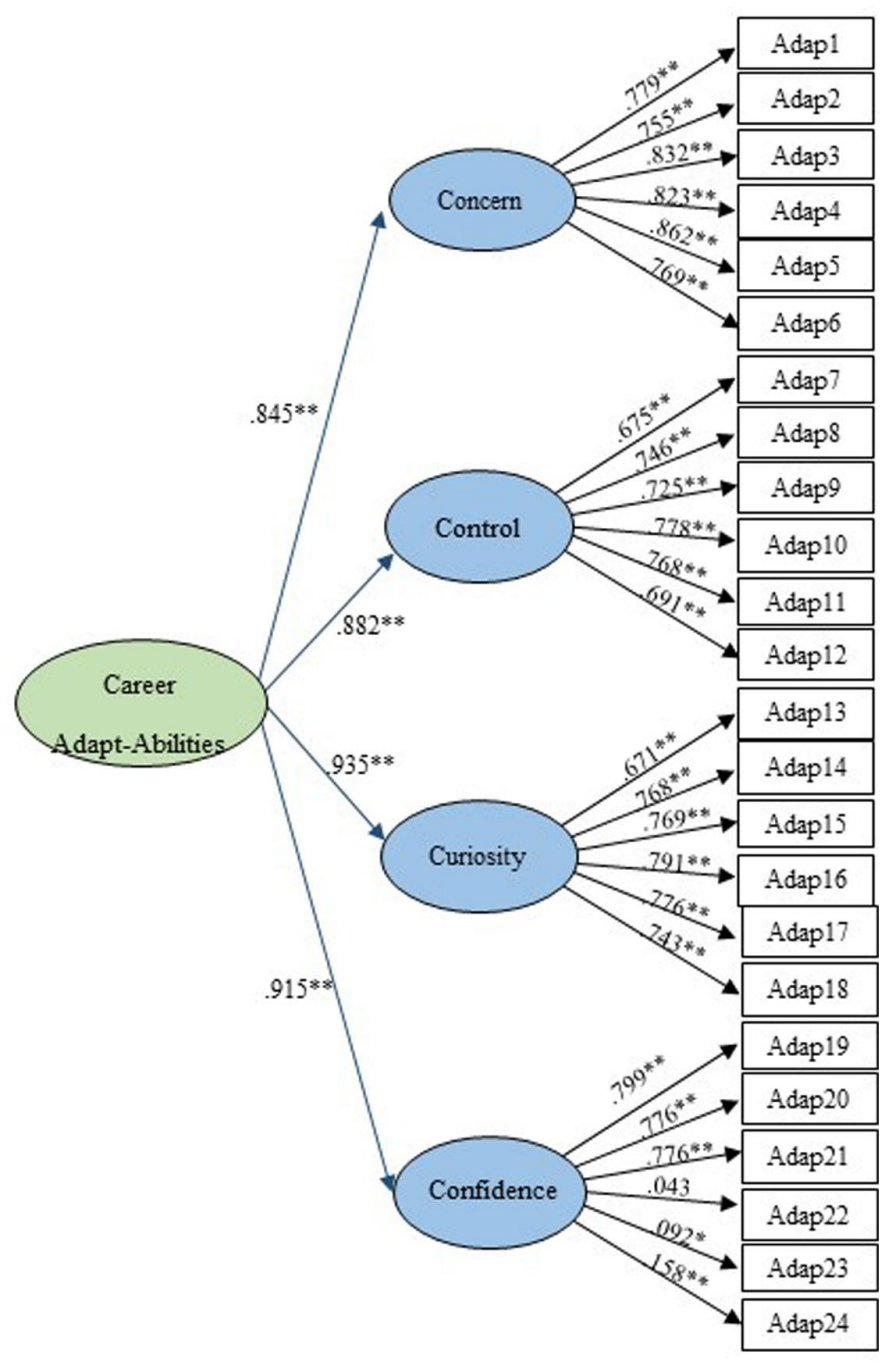
Second-order factor model.

In the first-order and second-order factor CFA models, all indices met the necessary criteria for assessing the four career adaptability dimensions. The standardized loadings from items to their respective factors ranged from 0.043 to 0.862, with coefficients from the first-order factors to the second-order career adaptabilities ranging from 0.845 to 0.935. Apart from item 22 (Adapt22), all loadings were significant at *p* < 0.001. The factor structure, as depicted in Figure 2, indicates robust psychometric properties of the scale within the current sample of Thai undergraduate students, with all items serving as strong indicators of the second-order constructs (Figure 3).

### 4.3 MIMIC model with DIF

**Figure 4:**
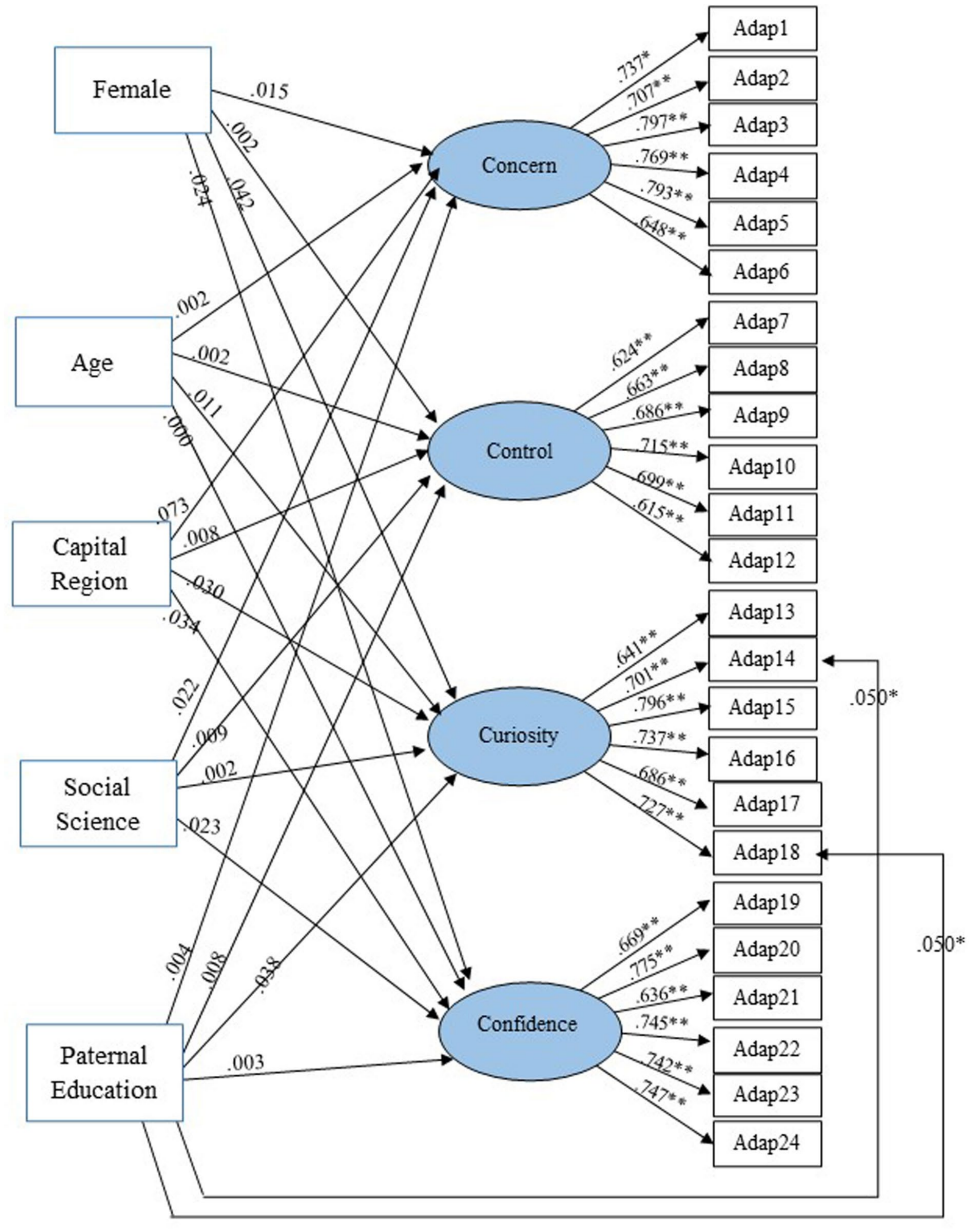
MIMIC model with DIF.

The MIMIC model with Differential Item Functioning (DIF) was employed to assess the impact of sociodemographic factors on the CAAS in its Thai version (CAAS-Thai). The model included a freely estimated direct effect from covariates to the CAAS-Thai items. Table 3 and Figure 4 illustrate that the model demonstrated a good fit with the data, as evidenced by various fit indices: χ^2^ (278) = 888.168, df = 230, p < 0.001, χ^2^/df = 3.195, CFI = 0.918, TLI = 0.883, RMSEA = 0.072 (95% CI: 0.066, 0.077). All measured factor loadings were statistically significant, affirming the reliability of the model.

In exploring sociodemographic differences using the MIMIC model with DIF on the four CAAS-Thai subscales, we investigated how covariates influenced both the overall CAAS score and specific item responses (Figure 4). While all measured factor loadings were statistically significant, interactions between the covariates and the latent factors were found to be statistically nonsignificant (*p* > 0.05). This indicates that students’ gender, age, university region, program of study, and paternal education level did not exert a significant direct effect on any of the four latent factors (concern, control, curiosity, and confidence), suggesting that career adaptability did not vary based on these sociodemographic characteristics.

However, noteworthy findings emerged concerning students whose fathers held a bachelor’s degree or higher (paternal education). These students demonstrated greater adaptability in specific aspects, notably in items Adap14 (Looking for opportunities to grow as a person: *β* = 0.050, *p* < 0.05) and Adap18 (Becoming curious about new opportunities: *β* = 0.050, *p* < 0.05). These nuances highlight the importance of considering parental education levels in understanding and fostering certain dimensions of career adaptability among undergraduate students.

## 5 Discussion

In this study, the exploration focused on the psychometric properties and construct validity of the CAAS through the analysis of a sample of Thai undergraduate students. The CAAS-Thai demonstrated robust psychometric properties, evident in the strong intercorrelations among its four subscales: concern, control, curiosity, and confidence. High scores on one subscale were consistently associated with high scores on the others, indicating internal coherence within the instrument. Importantly, our findings revealed that the overall scale and its subdimensions exhibited correlations comparable to those reported by Duangsao et al. (2021) in a study involving Thai undergraduate students from Bangkok. This consistency suggests that the CAAS-Thai maintains reasonable construct validity. Moreover, both the four-factor and second-order factor models displayed favorable fit indices, with χ^2^/df less than 3, RMSEA and SRMR less than 0.08, and CFI and TLI exceeding 0.90. These results align with previous studies by Savickas and Porfeli (2012) on the international form, Duangsao et al. (2021) in Thailand, Sulistiani et al. (2019) in Indonesia, Tien et al. (2014) in Macau, and Dries et al. (2012) in Belgium, supporting the applicability of the CAAS-international form with Thai students.

The MIMIC model was employed to investigate whether sociodemographic backgrounds influenced CAAS scores, revealing that none of the sociodemographic factors significantly impacted any of the four subscales. However, in the MIMIC model with Differential Item Functioning (DIF), paternal education emerged as the only sociodemographic factor with uniform DIF, exerting a significant effect on two specific outcomes: “looking for opportunities to grow as a person” and “becoming curious about new opportunities.” According to Montoya and Jeon (2019), a statistically significant path in a MIMIC model suggests that individuals with the same latent variable might perceive the item differently. In this context, this finding indicated that paternal education level played a role in shaping students’ curiosity related to career adaptability. Specifically, a father with a bachelor’s degree or higher positively influenced Thai university students’ inclination to explore and seek opportunities for personal development. This aligns with research by Zammitti et al. (2020), emphasizing the significance of curiosity in career exploration and decision-making.

Building on this, our study supports existing literature suggesting that parental support, demographic background, socioeconomic status, and parenting styles can influence career development and outcomes (Guan et al., 2018). In light of these diverse findings, we propose further research to explore the intricate ways in which parental involvement in their children’s education contributes to shaping their future careers. Understanding these dynamics could offer valuable insights for educational and career development interventions.

## 6 Limitations and future research

There are two significant limitations in this study that could be addressed in future research. First, because we conducted this study with a sample of Thai university students, the findings cannot be applied to the general population. Additionally, the students in our sample were pursuing different program majors and courses of study, so we did not incorporate internship experience in our survey. Future researchers should examine the relationships between CAAS scores and subscale scores and internship experience, work environments and university learning environments. All of these factors might also shape students’ career adaptability.

## 7 Conclusion

The study revealed that the CAAS-Thai and its components are reliable and highly interrelated, mirroring the performance of the CAAS-International. It suggests that CAAS-Thai is suitable for those assessing career adaptability in Thai undergraduates, particularly for predicting their job-seeking success post-graduation. This tool is also useful for career counseling and preparing students for future job challenges. Additionally, the study’s use of the MIMIC model with DIF highlights how Thai students’ sociodemographic factors influence their career adaptability. These insights are valuable for university administrators to enhance students’ adaptability skills, ultimately aiding in their smooth transition to the professional world.

## Data availability statement

The raw data supporting the conclusions of this article will be made available by the authors, without undue reservation.

## Ethics statement

The studies involving humans were approved by The Human Research Ethics Committee of Suranaree University of Technology. The studies were conducted in accordance with the local legislation and institutional requirements. The ethics committee/institutional review board waived the requirement of written informed consent for participation from the participants or the participants’ legal guardians/next of kin because All participants gave their informed consent verbally for inclusion before they participated in the study.

## Author contributions

BK: Data curation, Formal analysis, Funding acquisition, Investigation, Methodology, Project administration, Resources, Software, Supervision, Validation, Visualization, Writing – original draft, Writing – review & editing.
